# Latency of epileptic and psychogenic nonepileptic seizures

**DOI:** 10.1055/s-0043-1768160

**Published:** 2023-07-24

**Authors:** Hulya Ozkan, Meliha Turksever, Baburhan Guldiken, Necdet Sut

**Affiliations:** 1 Trakya University, School of Medicine, Department of Neurology, Edirne, Turkey.; 2 Trakya University, School of Medicine, Department of Biostatistics and Informatics, Edirne, Turkey.

**Keywords:** Psychogenic Nonepileptic Seizures, Epilepsy, Electroencephalography, Convulsiones Psicógenas no Epilépticas, Epilepsia, Electroencefalografía

## Abstract

**Background** Due to their semiological similarities, psychogenic nonepileptic seizures (PNESs) can occasionally hardly be differentiated from epileptic seizures (ESs), and long-term video-electroencephalographic monitoring (VEM) is needed for the differential diagnosis.

**Objective** To investigate the time of the first clinical event and its distribution on the days of VEM in ES and PNES patients.

**Methods** In total, a consecutive series of 48 PNES and 51 ES patients matched for gender and age were retrospectively and consecutively evaluated. The time distribution of the seizures during the day was noted. Seizure latency was determined as the time in hours from the start of the video-electroencephalographic recording to the first clinical event.

**Results** The seizure latency was significantly shorter in PNES patients compared to ES patients ( *p* < 0.001). Seventy-two percent of PNES patients and 49.1% of ES patients had their first seizure in the 24 hours of video-EEG recording ( *p* = 0.023). Recording longer than 48 hours was required for 12.5% of PNES patients and 37.3% of ES patients ( *p* = 0.006). While ESs were almost evenly distributed throughout the day, most PNESs occurred during the evening hours ( *p* = 0.011).

**Conclusion** We observed that the PNESs appeared earlier than the ESs in the VEM and were concentrated during daylight hours. Although not strictly reliable, seizure latency can contribute to the differential diagnosis of ES and PNES.

## Resumen

**Antecedentes** Debido a sus similitudes semiológicas, las crisis no epilépticas psicógenas (CNEP) en ocasiones apenas se pueden diferenciar de las crisis epilépticas (CE), y se necesita una monitorización video-electroencefalográfica (EEG) prolongada para el diagnóstico diferencial.

**Objectivo** Investigar el momento del primer evento clínico y su distribución en los días de monitorización video-EEG en pacientes con CE y CNEP.

**Métodos** Se evaluó retrospectivamente a una serie consecutiva de 48 pacientes con CNEP y 51 con ES emparejados por sexo y edad. Se anotó la distribución temporal de las incautaciones durante el día. La latencia de las crisis se determinó como el tiempo en horas desde el inicio de la grabación del video-EEG hasta el primer evento clínico.

**Resultados** La latencia de las crisis fue significativamente menor en los pacientes con CNEP en comparación con los pacientes con CE ( *p* < 0,001). El 72% de los pacientes con CNEP y el 49,1% de los pacientes con CE tuvieron su primera crisis en las 24 horas de registro del video-EEG ( *p* = 0,023). Se requirió un registro de más de 48 horas para el 12,5% de los pacientes con CNEP y el 37,3% de los pacientes con CE ( *p* = 0,006). Mientras que las CE se distribuyeron casi uniformemente a lo largo del día, la mayoría de las CNEP ocurrieron durante las horas después del anochecer ( *p* = 0,011).

**Conclusión** Observamos que las CNEPs aparecieron antes que las CEs en la monitorización video-EEG, y se agruparon durante las horas del día. Aunque no es estrictamente confiable, la latencia de las crisis puede contribuir al diagnóstico diferencial de ES y CNEP.

## INTRODUCTION

It is known that epileptic seizures (ESs) and psychogenic nonepileptic seizures (PNESs) can occasionally be hardly distinguished based only on semiology. 1 Most of the semiological signs, such as hyperventilation, crying, resistance to eyelid opening, and pelvic movements, are mostly specific to PNES but may be also encountered in ESs. Video EEG monitoring (VEM) is the gold standard for the differential diagnosis of PNES from ES. 2 Video EEG monitoring units are established mostly in tertiary health clinics, need a skilled epilepsy team, and are additionally used for the presurgical evaluation of drug resistant epilepsy patients. The time until seizures occur (latency) directly affects the time of the differential diagnosis. 3 It is an easily available quantitative value, but its contribution to the differential diagnosis of ES and PNES is not fully known. While some studies showed significant latency differences between PNES and ES, 3 4 5 other studies found no significant difference and no contribution to diagnosis. 6 7 8 9 However, these studies differed in terms of the groups examined (diagnostic, classification, preoperative evaluation), 7 10 recording times, 10 induction techniques used, 7 11 12 13 drug reduction/ discontinuation protocols, 7 10 14 PNES semiology, 10 15 and seizure frequency in the pre-VEM period. 6

In this study, we aimed to investigate the difference of the seizure latencies in ES and PNES patients and its contribution to the diagnosis.

## METHODS

The data of 497 adult (> 18 years old) patients hospitalized in our VEM unit for the differential diagnosis of ES, seizure classification, or pre-surgical evaluation over a 7-year period were retrospectively reviewed. Forty-eight PNES patients (48/497) who were definitively diagnosed in VEM, and 51 consecutive ES patients matched for gender and age were included in the study. A power value of 90% and an effect size of 0.687 was estimated with a total of 92 cases (46 ES and 46 PNES). Patients with other non-epileptic attacks (syncope, hypoglycemia, cardiac arrhythmia, cataplexy, and movement disorders) and 18 PNES patients with concomitant ES were excluded. A total of 294 seizures (40.1%; n = 118) PNES and 59.9% (n = 176) ES with focal ictal onset) were evaluated. According to their semiological features, PNES was grouped as subjective (29/118), akinetic (46/118), minor motor (26/ 118), and hypermotor (17/118) types. The day was divided into four equal intervals (06:01–12:00, 12:01–18:00, 18:01–24:00, and 24:01–06:00), and the time distribution of the seizures was determined. Patients' age, gender, medications and medications tapered during VEM, number of seizures recorded before and during VEM, and duration of monitoring were noted. Seizure latency was determined as the time in hours from the start of video EEG recording to the first seizure. Interictal and ictal EEG of PNES patients was normal. Neurological examinations and cranial imaging findings of the patients were recorded. In both groups, with the exception of standard provocative techniques (hyperventilation and intermittent photic stimulation), induction techniques such as saline injection and suggestion were not used. Antiepileptic drugs (AEDs) were reduced to induce seizures, and drugs were started again after the desired number of seizures if they were epileptic.

Our study was approved by the local Scientific Research Ethics Committee on April 24, 2021, with the decision number 09/10, and written informed consent was obtained from all participants prior to their inclusion in the study, which was conducted in accordance with the Declaration of Helsinki.

### Statistical analysis

Results are expressed as mean ± standard deviation or percentage. The compatibility of the qualitative data to normal distribution was examined using the Shapiro-Wilk test. The Mann-Whitney U test was used to compare qualitative values of the ES and PNES groups. Relationships between continuous variables were analyzed by using the Spearman correlation coefficient. The Pearson, Yates, or Fisher Chi-square tests were used to compare categorical data between the ES and PNES groups. Statistical analyzes were performed using the IBM SPSS Statistics for Windows, Version 20.0 (IBM Corp., Armonk, NY, USA) package program. A *P* -value < 0.05 was accepted as statistical significance limit value.

## RESULTS

There was no significant difference between the age and gender of PNES and ES patients. Age at the onset of seizure was lower in the ES group (20.7 ± 16.1 years vs. 27.5 ± 11.9 years, *p* < 0.001). Demographic and clinical features of the cases are summarized in Table 1 .

**Table 1:** Demographic and clinical features of all cases

		PNES group (n = 48)	ES group (n = 51)	*p*
Sex (n)	Female	32 (66.7%)	28 (54.9%)	0.304
	Male	16 (33.3%)	23 (45.1%)	
Age (year)		32.8 ± 11.7	36.9 ± 15.1	0.09
Age at the onset of seizures (year)		27.5 ± 11.9	20.7 ± 16.1	< 0.001
Family history of epilepsy (yes/no)		11/ 37	12/ 39	0.1
Number of AEDs before VEM (n)		1.6 ± 0.9	2.2 ± 1.2	< 0.001
Duration of AED use (year)		3.88 ± 4.99	11.37 ± 9.83	< 0.001
Duration of VEM (day)		4.5 ± 2.4	5.6 ± 2.8	< 0.001
Number of seizures before VEM/year		122.6 ± 164.7	123.5 ± 154.4	0.218
Number of seizures in VEM/day		1.0 ± 1.3	1.5 ± 3.5	< 0.001
Onset time of the AED reduction (day)		1.1 ± 1.3	1.8 ± 1.4	0.006
Seizure latency (hour)		20.4 ± 24.2	45.8 ± 54.9	< 0.001
Seizure in the first 24 hours (yes/no)		35/13	25/26	0.023
Seizure in the first 48 hours (yes/no)		42/6	32/19	0.006
Seizure after 48 hours (yes/no)		6/42	19/32	0.006

Abbreviations: AEDs, antiepileptic drugs; ES, epileptic seizure; PNES, psychogenic nonepileptic seizure; VEM, video-EEG monitoring.

Patients with PNES had a lower mean number of AEDs than ES patients and a shorter mean duration of AED use ( *p* < 0.001). The duration of VEM unit was shorter in PNES patients than in ES patients (4.5 ± 2.4 days vs. 5.6 ± 2.8 days, *p* < 0.001). There was no difference in the number of seizures per year before VEM among groups, but the mean number of seizures per day during VEM (seizures/ day) was higher in the ES group (1.5 ± 3.5 vs. 1.0 ± 1.3, *p* < 0.001).

**Figure 1:**
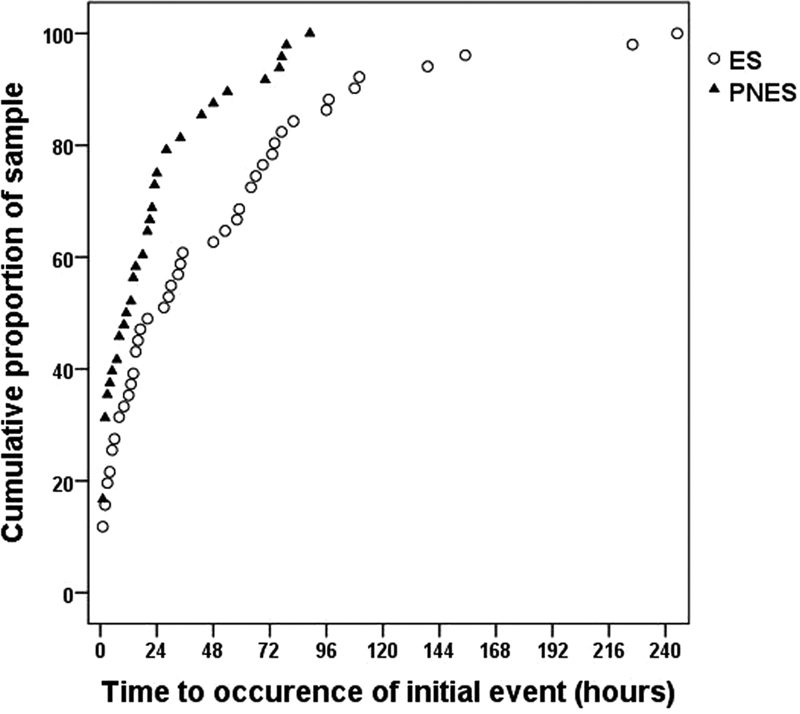
Cumulative probability plots of time to occurrence (in hours, following complete electrode placement) of first recorded event during admission, for patients with epileptic seizures and for patients with psychogenic nonepileptic seizures.

Seizure latency was shorter in PNES than in ES patients (20.4 ± 24.2 hour vs. 45.8 ± 54.9 hours, *p* < 0.001). The percentage of cases who had their first seizure within ≤ 24 hours of video-EEG recording was 72.9% in PNES and 49.1% in ES group ( *p* = 0.023). The difference in first recorded seizure persisted on the 2nd day in favor of PNES (87.5% of PNES patients vs. 62.7% of ES patients, *p* = 0.006), and recording longer than 48 hours for the 1st seizure was required in 12.5% of PNES vs. 37.3% of ES patients ( *p* = 0.006) ( Figure 1 ).

There was no difference between subjective, akinetic, minor motor, and hypermotor types of PNES in terms of seizure latencies ( Table 2 ).

**Table 2:** Demographic and clinical characteristics of PNES subgroups

Variables	Subjective (n = 29)	Akinetic (n = 46)	Minor motor (n = 26)	Hypermotor (n = 17)	p ^*^
Age (year)	26 (20–53)	37 (18–58)	38 (18–51)	26 (20–48)	0.771
Age at the onset of seizures (year)	21 (12–48)	32.5 (10–55)	23.5 (12–48)	22 (12–47)	0.784
Number of AEDs before VEM (n)	2 (0–3)	1 (0–3)	2 (1–3)	2 (0–2)	0.109
Duration of VEM (day)	4 (1–10)	3.5 (1–9)	5 (1–8)	4 (1–12)	0.307
Number of seizures in VEM/day	0.5 (0.2–1.5)	1 (0.2–5.0)	0.35 (0.1–7.0)	1 (0.2–1.7)	0.094
Onset time of the AED reduction (day)	1 (0–3)	0.5 (0–3)	0.5 (0–4)	1 (0–5)	0.863
Seizure latency (hour)	11 (1–70)	7.5 (1–89)	16.5 (1–79)	15.0 (1–77)	0.830

Abbreviations: AEDs, antiepileptic drugs; VEM, video-EEG monitoring.

Notes: Median (minimu –maximum); ^*^ Kruskal-Wallis test.

Most of the ESs and PNESs occurred between 06:01 and 24:00 ( Table 3 ). While the seizures of ES patients were almost equally distributed among all time intervals, most of the PNES cumulated especially between 18:01 and 24:00 (%35.6 vs. %22.2, *p <* 0.01). A total of 20.5% of ESs and 10.2% of PNESs appeared between 24:01 and 06:00 ( *p* = 0.029).

**Table 3:** Distribution of all seizures (n = 294) during the day

Time interval (hours)	Number of seizures		*p*
	PNES (n = 118)	ES (n = 176)	
06:01–12:00	26 (22.1%)	51 (28.9%)	0.184 ^**a**^
12:01–18:00	38 (32.2%)	50 (28.4%)	0.486 ^**a**^
18:01–24:00	42 (35.6%)	39 (22.2%)	< 0.01 ^a^
24:01–06:00	12 (10.2%)	36 (20.5%)	< 0.05 ^b^

Abbreviations: ES, epileptic seizure; PNES, psychogenic nonepileptic seizure.

Notes: n (%); ^a^ Pearson Chi-square test; ^b^ Yates Chi-square test.

## DISCUSSION

In our study, the percentage of the seizure occurrence in the first 24 and 48 hours were significantly higher in PNES patients than in ES patients, and the seizure latency was significantly shorter in PNES. Psychogenic nonepileptic seizures occurred more frequently between 18:01 and 24:00 and less frequently between 24:01 and 06:00 that ES.

Thirty-eight to 89.6% of patients that were hospitalized for differential diagnosis of ES had seizures within the first 24 hours in VEM. 3 4 6 7 8 9 The rate increased to 96.2% in the first 48 hours, and it was suggested that if induction techniques were to be used as a diagnostic tool, they could be avoided for the first 48 hours of VEM. 4 The rate of recording the seizure in the first 24 and 48 hours of VEM was higher in PNES than in ES. 3 4 A relatively shorter VEM (≤ 48 hours) was found to be sufficient for the diagnosis in patients with high clinical suspicion for PNES. 3

Similar to these studies, we found that 72.9% of PNES patients had their 1st seizure in the first 24 hours in contrast to 49.1% of ES patients ( *p* = 0.023). On the 2nd day of VEM, we saw that this difference increased even more between PNES and ES (87.5% vs. 62.7%, *p* = 0.006). Recording longer than 48 hours was required in only 25.3% of patients for the record of the 1st seizure. The mean seizure latency was found to be shorter in our PNES patients than in ES patients. This was in concordance with the study of Parra et al., which reported a shorter latency in PNES than in ES (15 ± 16.3 hours vs. 28.6 ± 34 hours, respectively). 4 Sagi et al. 3 found a shorter median latency in PNES patients than in ES patients (13.76 hours vs. 22.4 hours, respectively), but the mean latency showed no significant difference. In other studies, no significant relationship was found between the mean latencies of ES and PNES. 6 7 8

It is known that patients with PNES are prone to suggestion. Informing patients about the purpose of VEM may provoke PNES and may result in a shorter seizure latency in PNES. 13 14 It has been shown that a seizure while waiting in the outpatient clinic or during the examination is 75% predictive for PNES. 16 A patient who had a seizure while placing the EEG electrodes has a higher probability of suffering from PNES. 7 One of our PNES patients had a seizure within the first minute of the video EEG recording immediately after completing the electrode mounting procedure. The effects of hyperventilation and intermittent photic stimulation techniques on latency have been investigated in a few studies and their inducing effect has been shown especially in PNES. 11 12 13 The prolongation of hyperventilation to 5 minutes is effective in accelerating the occurrence of seizures. 12 Intravenous saline injection has been shown to induce the occurrence of PNES, but it is assumed not to be ethical because it may harm the patient-physician relationship and is not absolute sensitive. 17 18 All of our patients were informed about the purpose of VEM, but none of them underwent seizure induction, except for standard provocative techniques (3 minutes hyperventilation and intermittent photic stimulation). Several studies reported that motor and hypermotor types of PNES tend to occur earlier than other PNES types. 10 15 Because the pathophysiological brain mechanism of PNES is unclear, no explanation could be made for this finding. We found no difference in seizure latency among PNES types.

It was reported that there was a significant relationship between the frequency of seizures before VEM and the seizure latency of ES and PNES patients during the VEM. 6 In contrast, Eisenman et al. 14 did not find any significant relationship. The self-reported pre-VEM seizure numbers of our cases were similar, and no correlation was found between the seizure latency and the number of pre-VEM seizures.

Psychogenic nonepileptic seizures are not associated with sleep, while some ESs occur especially during sleep. 1 7 19 Nocturnal ESs are sometimes confused with PNESs due to the peculiar movements that may accompany them. Some PNESs seem to occur during sleep, but their EEG demonstrates wakefulness. 20 In our study, we found that PNES was not homogeneously distributed during the day and showed a diurnal pattern. A smaller proportion of patients with PNES had seizures between 24:01 and 06:00 than ES (10.2% vs. 20.5%, *p* = 0.029). These results are consistent with previous studies and show that seizures occurring between 24:01 and 06:00 are more likely to be ES than PNES. 19 21

While ES and PNES patients mostly have seizures in the first 48 hours of video-EEG monitoring, 3 4 5 7 8 9 10 15 22 35% of patients require follow-up longer than three days, and 7% longer than 1 week. 8 Monitoring longer than 5 days does not contribute additionally to the occurrence of PNES, and the inconclusive rate is higher than that of ES patients (28% vs. 12.5%). 23 In our study, the mean VEM duration of the PNES cases was 4.5 ± 2.4 days (min. 24 hours- max. 10 days) and 5.6 ± 2.8 days (min. 24 hours - max. 12 days) of the ES cases. Reducing or terminating the AEDs may occasionally induce seizures and may alter the length of VEM. In our study, the drug reduction protocol was decided by considering the clinical characteristics of cases.

### Limitations

Our study was a retrospective study, and the AED reduction protocol was not uniform among all patients and may influence the seizure latency. The positive aspects of our study are that spontaneous seizures were assessed without using induction techniques other than hyperventilation and photic stimulation, and the diagnosis of patients was made with the gold standard method VEM. Some PNES patients had nocturnal seizures and VEM documented wakefulness in these seizures.

In conclusion, our study demonstrated that seizure latency was significantly shorter in PNES than ES, and PNES clustered during daylight hours. Although not strictly reliable, seizure latency can also be considered in the differential diagnosis of ES and PNES.
